# Global QTL Analysis Identifies Genomic Regions on Chromosomes 4A and 4B Harboring Stable Loci for Yield-Related Traits Across Different Environments in Wheat (*Triticum aestivum* L.)

**DOI:** 10.3389/fpls.2018.00529

**Published:** 2018-04-25

**Authors:** Panfeng Guan, Lahu Lu, Lijia Jia, Muhammad Rezaul Kabir, Jinbo Zhang, Tianyu Lan, Yue Zhao, Mingming Xin, Zhaorong Hu, Yingyin Yao, Zhongfu Ni, Qixin Sun, Huiru Peng

**Affiliations:** State Key Laboratory of Agrobiotechnology, Key Laboratory of Crop Heterosis and Ultilization, The Ministry of Education, Key Laboratory of Crop Genetic Improvement, College of Agronomy and Biotechnology, China Agricultural University, Beijing, China

**Keywords:** stable QTL, yield components, plant height, heat susceptibility index, pleiotropy, marker-assisted selection, wheat

## Abstract

Major advances in wheat production are needed to address global food insecurity under future climate conditions, such as high temperatures. The grain yield of bread wheat (*Triticum aestivum* L.) is a quantitatively inherited complex trait that is strongly influenced by interacting genetic and environmental factors. Here, we conducted global QTL analysis for five yield-related traits, including spike yield, yield components and plant height (PH), in the Nongda3338/Jingdong6 doubled haploid (DH) population using a high-density SNP and SSR-based genetic map. A total of 12 major genomic regions with stable QTL controlling yield-related traits were detected on chromosomes 1B, 2A, 2B, 2D, 3A, 4A, 4B, 4D, 5A, 6A, and 7A across 12 different field trials with timely sown (normal) and late sown (heat stress) conditions. Co-location of yield components revealed significant tradeoffs between thousand grain weight (TGW) and grain number per spike (GNS) on chromosome 4A. Dissection of a “QTL-hotspot” region for grain weight on chromosome 4B was helpful in marker-assisted selection (MAS) breeding. Moreover, this study identified a novel QTL for heat susceptibility index of thousand grain weight (HSITGW) on chromosome 4BL that explains approximately 10% of phenotypic variation. *QPh.cau-4B.2, QPh.cau-4D.1* and *QPh.cau-2D.3* were coincident with the dwarfing genes *Rht1, Rht2*, and *Rht8*, and haplotype analysis revealed their pleiotropic architecture with yield components. Overall, our findings will be useful for elucidating the genetic architecture of yield-related traits and developing new wheat varieties with high and stable yield.

## Introduction

Common wheat (*Triticum aestivum* L.) is one of the most widely adapted food crops worldwide, providing approximately 30% of global grain production and 20% of the calories consumed by humans (FAO, 2017). The development of high-yield varieties is one of the important targets of modern wheat breeding programs worldwide because of the ever-growing global population and limited land for agricultural expansion (Lobell et al., 2011; Ray et al., 2012). Therefore, the identification, understanding and incorporation of QTL/genes that beneficially influence yield can facilitate the genetic improvement of varieties with high yield.

Grain yield in wheat is a complex quantitative trait that is strongly influenced by interacting genetic and environmental factors and can usually be broken down into three components: spikes per plant (SPP), grain number per spike (GNS), and thousand grain weight (TGW) (Quarrie et al., 2006; Gao et al., 2015). These yield components are sequentially fixed, influencing each other during the growth cycle, and are affected by other traits, such as plant height (PH), crop phenology, and biomass. Also they vary in terms of heritability (Del Moral et al., 2003; Ogbonnaya et al., 2017). SPP reflects wheat tiller capacity, which is one of the key characteristics affecting the yield potential of cereal crops (Naruoka et al., 2011). To date, wheat tillering QTL have been identified on chromosomes 1A, 1B, 1D, 2A, 2B, 2D, 3A, 3B, 4D, 5A, 5D, 6A, 6D, and 7A (Spielmeyer and Richards, 2004; Kuraparthy et al., 2007; Liu G. et al., 2014; Wang et al., 2016). Wheat spike is an important reproductive organ and is positively correlated with grain yield. Previous studies have identified more than 100 QTL for GNS distributed on all 21 chromosomes in wheat through linkage analysis and association analysis; these QTL were primarily located on chromosomes 1A, 1B, 1D, 2A, 2D, 3B, 3D, 4A, 5A, 6A, 7A, and 7D (Gao et al., 2015; Zhai et al., 2016; Cui et al., 2017; Shi et al., 2017; Zhou et al., 2017). Meanwhile, several genes related to GNS have been cloned and characterized using a homology-based approach (Zhang et al., 2011, 2015; Zheng et al., 2014). Grain weight is another essential yield component that is more stably inherited than final yield and is part of domestication syndrome in cereal crops (Quarrie et al., 2005; Meyer and Purugganan, 2013). Recently, several major QTL for grain size have been cloned and characterized in rice, providing important information about the molecular basis of grain weight in crop plants (Wang et al., 2008; Huang et al., 2013; Zuo and Li, 2014). Briefly, these cloned rice genes for grain size are mainly involved in multiple signaling pathways, including ubiquitination-mediated proteasomal degradation, phytohormones, and G protein signaling, to regulate cell division and cell expansion (Zuo and Li, 2014; Li and Yang, 2017). In wheat, a wealth of QTL for grain weight have been identified to date on almost all wheat chromosomes based on linkage mapping in bi-parental genetic populations and genome-wide association study (GWAS), and a number of genes associated with grain weight have been isolated using comparative genetic analysis (Quarrie et al., 2005; Zhang et al., 2010; Liu G. et al., 2014; Gao et al., 2015; Zanke et al., 2015; Li and Yang, 2017; Nadolska-Orczyk et al., 2017; Sajjad et al., 2017; Shao et al., 2017; Zhai et al., 2017). Nonetheless, to the best of our knowledge, there are no reports of cloned major QTL for grain weight through map-based cloning approach in wheat, and the molecular roles of QTL/genes in the regulation of grain weight are still largely unknown.

Wheat is a typical cool season crop, and increasing temperature is a major limitation to further improve wheat yield potential (Wardlaw et al., 1989; Acuna-Galindo et al., 2015; Ni et al., 2017). It was estimated that each temperature increase of one degree Celsius reduces grain weight per spike (GWS) by 3–4% (Wardlaw et al., 1989) and global wheat production by 6% (Asseng et al., 2015). Correspondingly, high temperatures can reduce GNS at the pre-flowering and flowering stages, and reduce grain weight at early grain filling stage (Sharma et al., 2016; Shirdelmoghanloo et al., 2016; Valluru et al., 2017). Therefore, the identification of stable and robust QTL for yield-related traits under varying severities of heat-prone environments is useful for maintaining wheat adaptability and production stability against a backdrop of fluctuating climate change patterns (Ogbonnaya et al., 2017). Moreover, heat-tolerant QTL mapping and the identification of traits associated with heat tolerance of yield components for common QTL are pre-requisites for developing molecular markers suitable for heat tolerance breeding (Shirdelmoghanloo et al., 2016). Despite its importance, only a few QTL mapping studies for the heat tolerance of yield components in wheat have been reported (Mason et al., 2010; Paliwal et al., 2012; Shirdelmoghanloo et al., 2016; Ni et al., 2017).

A breakthrough in wheat production during modern breeding was the utilization of reduced height (Rht) loci, which led to the Green Revolution in the late twentieth century (Hedden, 2003). To date, more than 20 dwarfing genes have been reported in wheat, and *Rht1* (*Rht-B1b*), *Rht2* (*Rht-D1b*), and *Rht8* are currently the three most commonly adopted dwarfing genes worldwide (Ellis et al., 2002; Zhang et al., 2006; Tian et al., 2017). The *Rht1* and *Rht2* belong to a group of genes known as gibberellic acid (GA) insensitive dwarfing genes and are located on chromosomes 4BS and 4DS, respectively (Peng et al., 1999; Achard et al., 2006). The two *Rht-1* homoeoloci, *Rht-B1* and *Rht-D1*, exert a pleiotropic effect on grain number, grain weight and yield in addition to reducing height (Zhang et al., 2013; Würschum et al., 2017). GA-responsive *Rht8*, located on chromosome 2DS, is another extensively used dwarfing gene, and it has been implemented in different environments because it has no effect on grain yield (Zhai et al., 2016; Tian et al., 2017). Furthermore, grain yield in wheat largely depends on plant architecture, particularly plant height (PH); thus, genetic loci associated with PH and yield components that are obtained by QTL mapping can provide a clear understanding of genetic relationships.

Recently, we developed a DH population derived from an elite cross of Nongda3338 (ND3338), and Jingdong6 (JD6) that exhibit contrasting phenotypes in PH, SPP, GNS, TGW, and GWS. Hence, the objectives of this study were to (i) evaluate the phenotypic performance of yield-related traits across different field trials with normal and late sowing mediated heat stress conditions; (ii) identify genomic regions with stable and robust QTL associated with yield-related traits; (iii) detect QTL controlling heat susceptibility index of thousand grain weight (HSITGW) for two contrasting treatments; and (iv) provide diagnostic markers to be deployed in marker-assisted selection (MAS) breeding for high-yield and heat-tolerant wheat varieties.

## Materials and methods

### Plant materials and field experiments

A DH population consisting of 203 individuals was developed through *in vitro* anther culture (De Buyser and Henry, 1980) of the F_1_ hybrids from a cross between two Chinese elite winter wheat varieties, ND3338 and JD6. Briefly, the female parent ND3338 is a “core parental” breeding line for the North China Winter Wheat Breeding Program with high general combining ability developed by China Agricultural University, while JD6 is a variety released by Beijing Academy of Agricultural and Forestry Sciences. The results of functional molecular markers (Ellis et al., 2002) indicated that ND3338 has mutant (dwarf) *Rht-B1b* and *Rht-D1b*, while JD6 possesses wild-type *Rht-B1a* and *Rht-D1a* (Kabir et al., 2015). Additionally, the diagnostic molecular marker Xgwm261 for *Rht8* was mapped on the short arm of chromosome 2D in our DH population with the 192-bp allele (*Rht8c*) from parent JD6.

The field experiments included two parts. First, the plants were grown at a conventional sowing time during the autumn across four different geographical locations in northern China: Beijing, Linfen, Shijiazhuang, and Urumqi. Dry-hot wind, defined as strong wind with high temperature and low humidity, often occurs during the grain filling period in the higher latitude areas of northern China (Teixeira et al., 2013; Liu B. et al., 2014; Ni et al., 2017). Second, two additional experiments were conducted under a timely sown condition and a late-sown condition to expose plants to higher temperatures (heat stress), particularly during grain filling period, at two locations in China: Linfen and Sanyuan. Detailed environment characteristics are provided in Table 1. The method of late-sown trials was as described by Cheng et al. (2015). Meteorological data for the experiment sites are presented in Table S1. In each field environment, the 203 DH lines and their parents were planted in randomized complete blocks with three replicates. Each plot contained two rows that were 2 m long and 30 cm apart with a sowing rate at 30 seeds in each row. All fields were well-watered by both rainfall and broad irrigation. Other management procedures of field trials followed local standard practices.

**Table 1:** Characteristics of the studied environments, period of the experiments and number of traits evaluated in each field trial.

**Growing season**	**Location**	**Code**	**Latitude**	**Longitude**	**Altitude(m)**	**Sowing date**	**Harvesting date**	**GWS^a^**	**PH^a^**	**SPP^a^**	**GNS^a^**	**TGW^a^**
2007–2008	Beijing	E1	39°48′N	116°28′E	31	2007/10/1	2008/6/15	√^b^	√	√	√	√
2007–2008	Linfen	E2	36°04′N	111°30′E	450	2007/10/4	2008/6/13	√	√	√	√	√
2007–2008	Shijiazhuang	E3	38°02′N	114°25′E	81	2007/10/2	2008/6/13	√	√	√	√	√
2008–2009	Beijing	E4	39°48′N	116°28′E	31	2008/10/1	2009/6/16	√	√	√	√	√
2008–2009	Linfen	E5	36°04′N	111°30′E	450	2008/10/5	2009/6/14	√	√	√	√	√
2008–2009	Shijiazhuang	E6	38°02′N	114°25′E	81	2008/10/2	2009/6/12	√	√	√	√	√
2011–2012	Urumqi	E7	43°47′N	87°39′E	836	2011/9/26	2012/7/10	√	√	√	√	√
2012–2013	Urumqi	E8	43°47′N	87°39′E	836	2012/9/30	2013/7/12	√	√	√	√	√
2013–2014	Linfen	E9	36°04′N	111°30′E	450	2013/10/3	2014/6/11	–	–	–	–	√
2013–2014	Sanyuan	E10	34°38′N	108°55′E	424	2013/10/5	2014/6/6	–	–	–	–	√
2014	Linfen	E11	36°04′N	111°30′E	450	2014/2/20	2014/6/30	–	–	–	–	√
2014	Sanyuan	E12	34°38′N	108°55′E	424	2014/3/2	2014/6/27	–	–	–	–	√

a *Trait abbreviations: plant height (PH), spikes per plant (SPP), grain number per spike (GNS), thousand grain weight (TGW) and grain weight per spike (GWS)*.

b *√ and - represent this trail has phenotypic data and no data, respectively*.

### Phenotypic evaluation and statistical analysis

Once the plants reached physiological maturity, 10 representative plants per genotype from each replication were used for phenotypic evaluation. PH (cm) was measured from the soil surface to the tip of the spike, excluding awns; SPP was also measured before harvesting. GNS, GWS, and TGW were measured after the seeds had naturally dried following harvest. Based on the TGW of two sowing dates, the HSI for each individual line was calculated using the formula by Fisher and Maurer (Fischer and Maurer, 1978):

$$\begin{array}{l} HSI=\left(1-{\text{Y}}_{\text{heatstress}}/{\text{Y}}_{\text{control}}\right)/\left(1-{\text{X}}_{\text{heatstress}}/{\text{X}}_{\text{control}}\right). \end{array}$$

where *Y*_heatstress_ and *Y*_control_ are the TGW means for each genotype under heat-stressed and controlled conditions, respectively, and *X*_heatstress_ and *X*_control_ are the TGW means for all lines under heat-stressed and controlled conditions, respectively.

Basic statistical analyses, phenotypic correlation and Shapiro-Wilk tests for departure from normality were performed by SPSS software version 20.0 (SPSS, Chicago, USA). The adjusted mean (Best Linear Unbiased Prediction, BLUP) values across multiple environments were calculated using SAS v9.1.3 (SAS Institute Inc., North Carolina, USA) with the PROC MIXED procedure. Broad sense heritability (*h*^2^) on a family basis was calculated with the PROC GLM procedure in SAS according to the following formula: *h*^2^ = σ_*g*_^2^/(σ_*g*_^2^+σ_*ge*_^2^*/n*+σ^2^*/nr*), where σ_*g*_^2^ is the genotypic effect, σ_*ge*_^2^ is the genotype by environmental effect, σ^2^ is the residual error, *n* is the number of environments and *r* is the number of replicates (Liu G. et al., 2014; Zhai et al., 2016). Linear regression analysis was conducted based on the additive effects in Microsoft Excel 2016. The biplot of principal component analysis (PCA) and additive main effects and multiplicative interactions (AMMI) analysis were performed in R software (v. 3.4.2) (R Core Team, 2013; Dixit et al., 2014).

### Genotyping and construction of genetic map

Young leaf tissues of the parents and DH lines at the seedling stage were used for total genomic DNA extraction with the modified CTAB method (Cheng et al., 2015). Approximately 2,800 SSR and STS markers were used to detect polymorphisms between the two parents. Primer sequences for most SSR markers are publicly available at http://wheat.pw.usda.gov/GG2/index.shtml. The PCR system, DNA amplification condition and product fragment detection were determined as described by Liu G. et al. (2014). In addition, the two parents and 203 DH lines were also genotyped with the Illumina 90K iSelect wheat SNP assay (Wang et al., 2014) at the Genome Center at the University of California, Davis. SNP clustering and genotype calling were performed using GenomeStudio version 2011.1 software (Cavanagh et al., 2013). The integrated genetic map based on SSR and STS markers, SNP makers and gene specific markers were generated using the programs RECORD 2.0 (Van Os et al., 2005) and JoinMap 4.0 (Van Ooijen, 2006). SNP markers with large numbers of missing values (>20%) were discarded. The map construction procedure is based on the methodology described by Zhai et al. (2016). Genetic linkage maps were compared with consensus 90K SNP maps (Wang et al., 2014) to orient each linkage group with respect to the short (S) and long (L) chromosome arms, further checking the accuracy of the marker order. Additionally, genetic maps and QTL graphs were drawn using MapChart 2.2 software (Voorrips, 2002).

### QTL detection

Mean data of each trait for individual environments and the adjusted mean (BLUP) values across multiple environments were used for QTL analysis with Windows QTL Cartographer software version 2.5 (Wang et al., 2012) through composite interval mapping (CIM). In QTL Cartographer, the parameters were as follows: model 6 (standard model), forward and backward regression, five control markers (co-factors), window size of 10 cM, and walk speed of 1 cM. An empirical genome-wide LOD threshold to identify significant QTL were calculated using 1,000 permutations for *P* ≤ 0.05. Confidence intervals were estimated based on positions ± 2 LOD (from the peak) method using QTL Cartographer. QTL with overlapping confidence intervals or QTL located within 10 cM region were considered equivalent. Only QTL that were significant at a LOD value ≥ 2.5 were accepted in this study. QTL names were denoted according to the International Rules of Genetic Nomenclature (http://wheat.pw.usda.gov/ggpages/wgc/98/Intro.htm). QTL for traits that co-localized within the same genomic region were assigned a common QTL name.

### Bioinformatics analysis

The SNP flanking sequences mapped in the integrated genetic map were aligned with respect to the newly released bread wheat Chinese Spring Reference sequence by International Wheat Genome Sequencing Consortium (IWGSC) (http://www.wheatgenome.org/, IWGSC RefSeq v1.0) and the coding sequences (CDS) with high-confidence genes to obtain physical positions and candidate genes. In addition, the annotation of high-confidence genes was obtained from wheat genome database websites (https://wheat-urgi.versailles.inra.fr/Seq-Repository/Annotations and http://plants.ensembl.org/Triticum_aestivum/Info/Index). Synonymous or nonsynonymous SNPs were annotated, and synteny analyses with rice genomes were performed as described by Wang et al. (2014).

## Results

### High-density integrated genetic linkage map construction

A coarse-scale linkage map was initially constructed over the whole wheat genome with 475 SSR and STS markers using 203 DH lines. To construct linkage map for the unlinked regions in the initial map, 81,587 SNPs from the wheat 90K SNP array were used for genotyping the ND3338/JD6 DH population (Wang et al., 2014), and 10,409 (12.76%) SNP markers that showed polymorphism between the parents were used for linkage analysis. Forty-six of these SNP markers were discarded because they had more than 20% missing data, and 176 SNP markers were not anchored on the linkage map. Finally, a high-density integrated genetic linkage map based on 475 SSR and STS markers, 10,187 transcript-derived SNP markers and two gene functional markers (*Rht1* and *Rht2*) was constructed for QTL mapping (Tables S3, S4). The 10,664 markers represented a total of 2,017 unique loci (18.91%) distributed among 26 linkage groups (LGs) representing the 21 hexaploid wheat chromosomes (Tables S3–S5). All linkage maps covered 3,391.20 cM in length with an average density of 1.68 cM/locus, and the map length of the three A, B, and D genomes was divided approximately equally (Table S3). However, the distribution of markers in the genomes was not uniform with about five times as many polymorphic markers mapping to the A and B genomes than to the D genome, namely, 4,692, 4,530, and 965 markers for the A, B, and D genomes, respectively (Table S3). In total, 958 (47.50%) of the 2,017 unique loci in the ND3338/JD6 integrated linkage map had segregation ratios that deviated significantly (chi-square ≤ 0.05) from the expected 1:1 ratio (Table S5). Of these loci, 527 (55.01%) favored the male parent (JD6), and 431 (44.99%) loci favored the female parent (ND3338).

### Phenotypic performance across multi-environments

**Figure 1:**
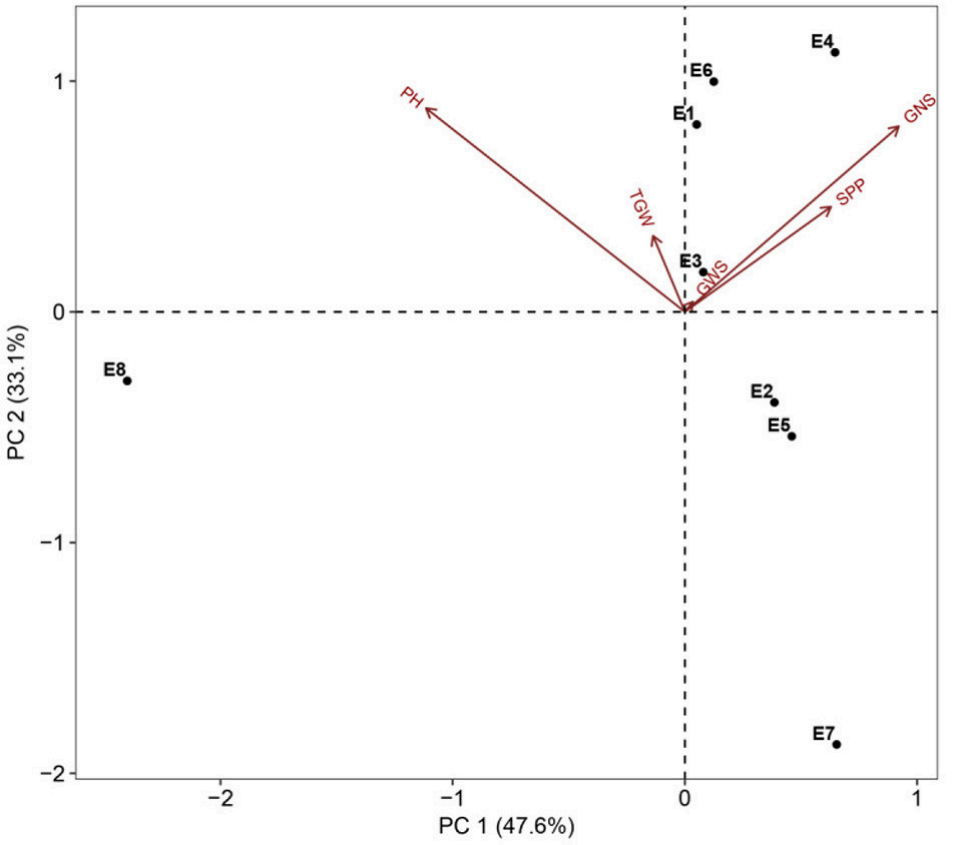
PCA biplot for environmental variability prevailing in the eight shared experimental sites in terms of plant height (PH), spikes per plant (SPP), grain number per spike (GNS), thousand grain weight (TGW), and grain weight per spike (GWS). Codes for the sites are explained in Table 1.

Two parents and 203 DH lines were trialed in a multi-environment design, including different locations and growing seasons, to identify stable QTL for yield-related traits across different field trials with timely sown (normal) and late sown (heat stress) conditions (Table 1). The DH population means and ranges of five yield-related traits (PH, SPP, GNS, TGW, and GWS) across the eight shared environments are shown in Table S2. JD6 had higher PH, TGW, and GWS and lower SPP and GNS than ND3338 (Table 2, Figure S1). PH, SPP, TGW, and GWS displayed obvious deviations from normality in the population, whereas GNS exhibited normal distributions with the BLUP values of eight shared environments (Table 2). All traits had *h*^2^ over 0.80, and the highest *h*^2^ was observed for PH, which reached 0.99, suggesting that PH was controlled by major effect genes in the DH population (Table 3). Pearson's correlation coefficients of the five traits were estimated based on the BLUP values of eight shared environments, which showed that SPP was significantly and negatively corrected with PH, GNS, TGW, and GWS (Table 4). TGW displayed strongly positive correlations with PH and GWS and a significantly negative correlation with GNS (Figure 1, Table 4). ANOVA with the eight shared environments for five yield-related traits revealed that there were significant variations from the environments and DH lines by environment interactions (Table 3). PCA biplot for environmental variability showed the differences among the eight shared experimental sites (Figure 1). For contrasting field trials, means of TGW in the DH population showed significant reduction under the late-sown heat-stressed condition compared to the timely sown condition, which was consistent with meteorological data (Figure S2, Table S1). Meanwhile, the HSI of TGW was calculated to assess the heat tolerance performance of the DH population, which suggested that a part of the progeny had transgressive phenotypes and the DH lines showed a continuous normal distribution based on the Shapiro-Wilk test (Figure S2).

**Table 2:** Parental and population means, ranges and the Shapiro-Wilk test for plant height (PH), spikes per plant (SPP), grain number per spike (GNS), thousand grain weight (TGW), and grain weight per spike (GWS) based on the adjusted mean (BLUP) values of eight shared environments.

**Trait**	**Parental lines**		**DH lines**					**The Shapiro-Wilk test**
	**ND3338**	**JD6**	**Minimum**	**Maximun**	**Mean**	**Median**	**Standard deviation**	**Significance**
PH	53.89	80.14	42.62	102.40	70.19	69.98	14.14	0.00
SPP	10.37	8.17	8.14	12.80	9.91	9.87	0.82	0.00
GNS	45.26	41.43	32.19	55.71	44.62	45.08	4.53	0.47
TGW	42.76	56.96	36.57	61.52	48.08	48.14	4.80	0.04
GWS	1.92	2.40	1.58	2.75	2.22	2.24	0.23	0.02

**Table 3:** Analyses of variance (ANOVA) and broad sense heritability estimates (*h*^2^) for plant height (PH), spikes per plant (SPP), grain number per spike (GNS), thousand grain weight (TGW), and grain weight per spike (GWS) based on eight shared environments in the ND3338/JD6 doubled haploid (DH) population.

**Source of variance**	**df**	**Sum of squares**				
		**PH**	**SPP**	**GNS**	**TGW**	**GWS**
Environments	7	106947.01^**^	48382.30^**^	87051.16^**^	33761.44^**^	210.57^**^
Replicates/environments	16	2273.75^**^	871.16^**^	919.01^**^	1690.11^**^	7.19^**^
Lines	202	969788.75^**^	4892.43^**^	115320.24^**^	146605.38^**^	314.87^**^
Lines × environments	1,414	34075.46^**^	6347.42^**^	59530.85^**^	27097.89^**^	202.31^**^
Error	3,232	35371.13	9568.23	74387.45	23111.16	201.31
Broad-sense heritability		0.99	0.83	0.93	0.97	0.91

** *Indicate significance at P ≤ 0.01 (2-tailed)*.

**Table 4:** Coefficients of correlation between plant height (PH), spikes per plant (SPP), grain number per spike (GNS), thousand grain weight (TGW), and grain weight per spike (GWS) based on the adjusted mean (BLUP) values of eight shared environments in the ND3338/JD6 doubled haploid (DH) population.

**Trait**	**PH**	**SPP**	**GNS**	**TGW**
SPP	−0.48^**^			
GNS	−0.09^**^	−0.29^**^		
TGW	0.64^**^	−0.37^**^	−0.44^**^	
GWS	0.51^**^	−0.60^**^	0.52^**^	0.52^**^

** **Indicate significance at P ≤ 0.01 (2-tailed).

### QTL mapping analysis

In this study, a total of 226 QTL controlling five yield-related traits and the heat susceptibility index (HSI) were detected across 12 different environments using CIM (Tables S6–S8). QTL that were repeatedly detected in ≥ 3 individual environments and in the BLUP analysis were considered to be stable. According to this criterion, 50 stable QTL for PH, SPP, GNS, TGW, and GWS were identified; and of these, 39 stable QTL were mapped within 12 genomic regions with corresponding physical intervals of Chinese Spring RefSeq v1.0 sequence on chromosomes 1B, 2A, 2B, 2D, 3A, 4A, 4B, 4D, 5A, 6A, and 7A (Table 5, Table S6). The other 168 putative QTL for five yield-related traits are listed in Table S7, and 8 QTL for the HSI of TGW were identified in two conditions (Table S8). Detailed parameters of QTL detected for each trait and environment are as follows.

**Table 5:** Genomic regions harboring stable QTL for plant height (PH), spikes per plant (SPP), grain number per spike (GNS), thousand grain weight (TGW), and grain weight per spike (GWS) in the ND3338/JD6 doubled haploid (DH) population.

**Chromosome**	**Genetic interval (cM)^a^**	**Physical distance (Mb)^b^**	**Traits^c^**	**Included QTL^d^**	**Detected environment^e^**	**References**
Chr.1B.1	31.2–42.0	598.43–641.84	PH (J)	***QPh.cau-1B.1***	E1/E2/E3/E4/E5/E6/E7/E8/C	Griffiths et al., 2012
			TGW(J)	*QTgw.cau-1B.1*	E2/E4/C	Cui et al., 2014
			SPP (N)	*QSpp.cau-1B.2*	E7	
			GWS (J)	*QGws.cau-1B.1*	E1	
Chr.2A	112.9–133.8	639.07–733.92	TGW (J)	***QTgw.cau-2A.3***	E2/E5/E6/E10/C	Cui et al., 2014
			SPP (N)	*QSpp.cau-2A.1*	E2/E6/C	
			GWS (J)	*QGws.cau-2A.3*	E1/E5/E7	
			GWS (J)	***QGws.cau-2A.2***	E1/E2/E5/C	
Chr.2B	111.6–131.9	177.65–657.73	GNS (N)	***QGns.cau-2B.4***	E1/E2/E4/E7/C	Liu G. et al., 2014
			GWS (N)	***QGws.cau-2B.1***	E2/E5/E8/C	Liu G. et al., 2014
			GWS (N)	***QGws.cau-2B.2***	E2/E3/E8/C	
			SPP (J)	*QSpp.cau-2B.5*	E4	
Chr.2D.1	26.9–69.8		PH (N)	***QPh.cau-2D.2***	E1/E2/E3/E4/E5/E6/E7/E8/C	
			PH (N)	***QPh.cau-2D.3***	E1/E2/E3/E4/E5/E6/E7/E8/C	Wu et al., 2010;Zhai et al., 2016
			TGW (N)	***QTgw.cau-2D.1***	E5/E6/E7/C	Cui et al., 2014
			GNS (N)	*QGns.cau-2D.1*	E4	
			GWS (N)	*QGws.cau-2D.1*	E4	
Chr.3A	65.8–105.8	705.31–749.11	PH (J)	***QPh.cau-3A.1***	E1/E2/E3/E4/E5/E6/E7/E8/C	Zanke et al., 2014
			PH (J)	***QPh.cau-3A.2***	E1/E2/E3/E4/E5/E7/E8/C	
			PH (J)	***QPh.cau-3A.3***	E1/E2/E3/E4/E5/E6/E7/E8/C	
			GNS (N)	*QGns.cau-3A.2*	E6	
Chr.4A	84.6–108.5	622.19–685.00	GNS (N)	*QGns.cau-4A.3*	E1/E2/E3/E6/E7	
			GNS (N)	***QGns.cau-4A.4***	E2/E3/E4/E5/E6/E7/E8/C	Gao et al., 2015;Cui et al., 2017
			TGW (J)	*QTgw.cau-4A.2*	E1/E4/E6/E8	Cui et al., 2014
			TGW (J)	***QTgw.cau-4A.3***	E1/E2/E3/E4/E6/E7/E8/E9/E11/E12/C	Gao et al., 2015;Cui et al., 2016
			GWS (N)	*QGws.cau-4A.3*	E5	
			GWS (N)	*QGws.cau-4A.4*	E5	
			SPP (J)	*QSpp.cau-4A.3*	E6	
Chr.4B	22.3–95.8	13.98–567.18	TGW (J)	***QTgw.cau-4B.1***	E2/E3/E5/E6/E8/E9/E10/C	
			TGW (J)	***QTgw.cau-4B.2***	E2/E3/E5/E6/E8/E9/E10/C	Chen et al., 2014
			TGW (J)	***QTgw.cau-4B.3***	E1/E3/E4/E5/E6/E7/E8/E12/C	Liu G. et al., 2014;Kumar et al., 2016
			TGW (J)	***QTgw.cau-4B.4***	E3/E4/E5/E7/E8/E11/E12/C	Liu G. et al., 2014;Kumar et al., 2016
			TGW (J)	*QTgw.cau-4B.5*	E4/E8/E11	Kumar et al., 2016
			SPP (N)	***QEp.cau-4B.3***	E4/E6/E7/E8/C	Liu G. et al., 2014
			SPP (N)	***QEp.cau-4B.4***	E1/E4/E6/E7/E8/C	Cui et al., 2014;Liu G. et al., 2014
			PH (J)	***QPh.cau-4B.2***	E1/E2/E3/E4/E5/E6/E7/E8/C	Wu et al., 2010;Gao et al., 2015
			GWS (J)	***QGws.cau-4B.3***	E1/E5/E6/E7/C	Liu G. et al., 2014
			GWS (J)	*QGws.cau-4B.4*	E3/E4/C	Liu G. et al., 2014
			GWS (J)	*QGws.cau-4B.5*	E3/E4/C	
			GWS (J)	*QGws.cau-4B.6*	E4	
Chr.4D	22.3–62.0	12.77–62.47	PH (J)	***QPh.cau-4D.1***	E1/E2/E3/E4/E5/E6/E7/E8/C	Liu G. et al., 2014;Gao et al., 2015
			PH (J)	***QPh.cau-4D.2***	E2/E3/E5/E8/C	
			SPP (J)	***QEp.cau-4D.1***	E1/E3/E4/C	
			TGW (J)	*QTgw.cau-4D.2*	E1/E3/E4/E5/E7/E8	Liu G. et al., 2014
			TGW (J)	*QTgw.cau-4D.3*	E4	
Chr.5A	23.2–63.9	11.05–460.52	GNS (J)	***QGns.cau-5A.1***	E3/E6/E7/C	
			GNS (J)	***QGns.cau-5A.2***	E1/E2/E3/E4/E6/C	Cui et al., 2014
			TGW (N)	***QTgw.cau-5A.2***	E3/E4/E5/E11/C	Gao et al., 2015;Wu et al., 2015
			TGW (N)	*QTgw.cau-5A.3*	E5/E11	Wu et al., 2015
			GWS (J)	*QGws.cau-5A.1*	E7	
Chr.6A	71.5–79.0	38.43–596.59	TGW (J)	*QTgw.cau-6A.5*	E3/E4/E5/E7	Gao et al., 2015;Tian et al., 2017
			TGW (N)	***QTgw.cau-6A.4***	E3/E6/E7/E12/C	
			PH (J)	***QPh.cau-6A.2***	E1/E2/E3/E4/E5/E6/E7/E8/C	Tian et al., 2017;Würschum et al., 2017
			GWS (N)	*QGws.cau-6A.4*	E8	
			GNS (N)	*QGns.cau-6A.2*	E1/E8	
Chr.7A	39.5–61.9	7.13–612.38	GNS (J)	***QGns.cau-7A.3***	E1/E2/E3/E5/E8/C	Quarrie et al., 2006
			GNS (J)	***QGns.cau-7A.2***	E1/E2/E3/E8/C	Quarrie et al., 2006; Zhai et al., 2017
			GNS (J)	***QGns.cau-7A.4***	E1/E3/E8/C	Quarrie et al., 2006
			GWS (J)	***QGws.cau-7A.2***	E2/E7/E8/C	
			PH (J)	***QPh.cau-7A.6***	E4/E5/E6/E8/C	Wu et al., 2010
			PH (J)	*QPh.cau-7A.5*	E1	
			SPP (N)	*QSpp.cau-7A.1*	E4	
Chr.7A	90.9–121.7	672.03–719.57	TGW (N)	***QTgw.cau-7A.2***	E2/E6/E9/C	Quarrie et al., 2006
			TGW (N)	***QTgw.cau-7A.3***	E2/E6/E9/E10/E11/C	Quarrie et al., 2006
			TGW (N)	***QTgw.cau-7A.4***	E2/E6/E9/E11/C	Quarrie et al., 2006
			SPP (N)	*QSpp.cau-7A.2*	E2/E3	Quarrie et al., 2006
			GNS (J)	***QGns.cau-7A.5***	E5/E6/E7/C	Quarrie et al., 2006
			GNS (J)	*QGns.cau-7A.6*	E2/E5	Quarrie et al., 2006

a *Additional details regarding the SNP markers within each QTL region can be found in Tables S6, S7*.

b *The corresponding physical distances (Mb) of the QTL regions on chromosomes 1B.1, 2A, 2B, 2D.1, 3A, 4A, 4B, 4D, 5A, 6A, and 7A were obtained by blasting the flanking sequences of SNP markers to the Chinese Spring RefSeq v1.0 sequence (Table S4)*.

c *The traits include plant height (PH), spikes per plant (SPP), grain number per spike (GNS), thousand grain weight (TGW), and grain weight per spike (GWS). The letters within the brackets indicate the origin of the increasing alleles with “N” and “J” representing ND3338 and JD6, respectively*.

d *QTL shown in bold are stable QTL that were detected in ≥3 individual environments and the BLUP analysis*.

e *C indicates the combined QTL analysis based on BLUP values*.

### Grain weight per spike (GWS)

**Figure 2:**
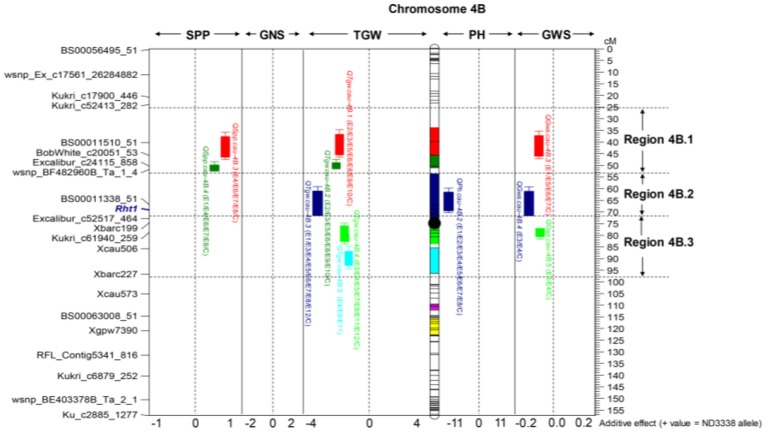
Dissection of the “QTL-hotspot” region on chromosome 4B for yield-related traits in the ND3338/JD6 DH population. Black dot represents the approximate centromere position. Different colored intervals on the chromosome indicate the corresponding QTL confidence intervals. The purple-colored and yellow-colored intervals indicate the confidence intervals of *QHsitgw.cau-4B.1* and *QHsitgw.cau-4B.2*, respectively. The representative makers and centiMorgan (cM) scale are shown on the left and right, respectively.

For GWS, a total of 47 QTL were identified across eight shared environments with a LOD score range of 2.52–15.23, explaining 2.98–24.17% of the variance (Tables S6, S7). Of these 47 QTL, 7 stable QTL were expressed in multiple environments on chromosomes 2A, 2B, 2D, 4B, and 7A (Table 5, Table S6). The major stable QTL for GWS was observed on chromosome 4B (*QGws.cau-4B.3*) with phenotypic variations of as much as 20.89%, and JD6 contributed the increasing allele (Figure 2, Table S6).

### Spikes per plant (SPP)

For SPP, a total of 35 QTL were identified across eight shared environments with a LOD score range of 2.54–13.62, explaining 2.98–18.47% of the variance (Tables S6, S7). Of these QTL, 4 stable QTL were mapped on chromosomes 4A, 4B, and 4D, which were designated *QSpp.cau-4A.1, QSpp.cau-4B.3, QSpp.cau-4B.4*, and *QSpp.cau-4D.1*, respectively (Figure 2, Table 5, Table S6). The alleles for increased SPP at four loci were all contributed by ND3338.

### Grain number per spike (GNS)

For GNS, a total of 34 QTL were identified across eight shared environments with a LOD score range of 2.51–13.48, explaining 3.13–21.36% of the variance (Tables S6, S7). Of these QTL, 9 stable QTL were located on chromosomes 2B, 4A, 5A, 6B, and 7A. The genomic regions on chromosomes 2B and 4A covered two stable QTL for GNS (*QGns.cau-2B.4* and *QGns.cau-4A.4*) with the superior alleles all coming from ND3338 (Table 5, Table S6). The highest phenotypic variation (up to 21.36%) for GNS was explained by *QGns.cau-4A.4*. The regions on chromosome 5A and 7A contained linked stable QTL for GNS (*QGns.cau-5A.1* and *QGns.cau-5A.2, QGns.cau-7A.2, QGns.cau-7A.3*, and *QGns.cau-7A.4*, respectively), where JD6 contributed the increasing allele (Table 5, Table S6). Both regions together explained 26.97% of the observed variation of GNS in the analysis of BLUP data.

### Thousand grain weight (TGW)

**Figure 3:**
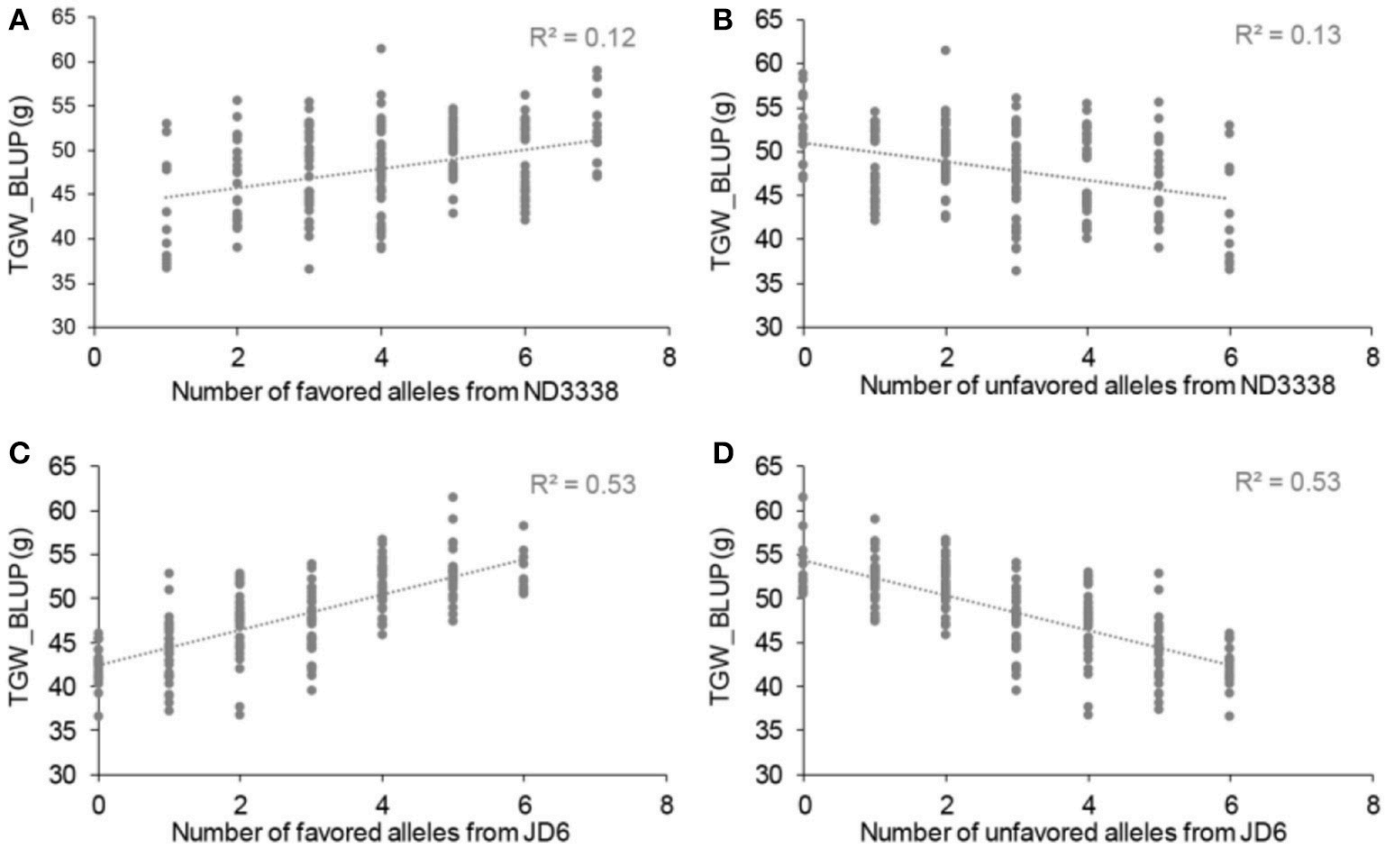
Allelic effects of favored and unfavored alleles from ND3338 **(A,B)** and JD6 **(C,D)** for thousand grain weight (TGW) based on linear regression.

For TGW, a total of 69 QTL were identified in 12 environments that include eight shared environments, two timely sown controlled environments and two late-sown heat-stressed environments with a LOD score range of 2.51–32.07, explaining 1.96–48.84% of the variance (Tables S6, S7). Of these, 13 stable QTL for TGW were mapped on chromosomes 2A, 2D, 4A, 4B, 5A, 6A, and 7A, and the favorable alleles were contributed by both parents, ND3338 and JD6 (Tables 5, 7, Table S6), which was consistent with regression analysis (Figure 3). The genomic regions on chromosomes 4B contained four adjacent major QTL for TGW (*QTgw.cau-4B.1, QTgw.cau-4B.2, QTgw.cau-4B.3*, and *QTgw.cau-4B.4*) with the superior alleles all coming from JD6, which together explained 47.23% of the total variation of TGW in the analysis of BLUP data (Figure 2, Table 5, Table S6). Similarly, the genomic regions on chromosome 7A contained three linked stable QTL (*QTgw.cau-7A.2, QTgw.cau-7A.3*, and *QTgw.cau-7A.4*) with the favored alleles all coming from ND3338, which together explained 22.72% of the observed variation of TGW in the analysis of BLUP data (Table 5, Figure 3, Table S6). Additionally, two stable QTL on chromosomes 4A and 4B, *QTgw.cau-4A.3* and *QTgw.cau-4B.4*, were constitutively expressed across both heat-stressed environments (Table 5, Table S6), and 5 QTL (*QTgw.cau-1B.3, QTgw.cau-1B.6, QTgw.cau-5B.2, QTgw.cau-6D.2*, and *QTgw.cau-7A.5*) were exclusively identified in the two heat-stressed trials (Table S7).

### Plant height (PH)

**Figure 4:**
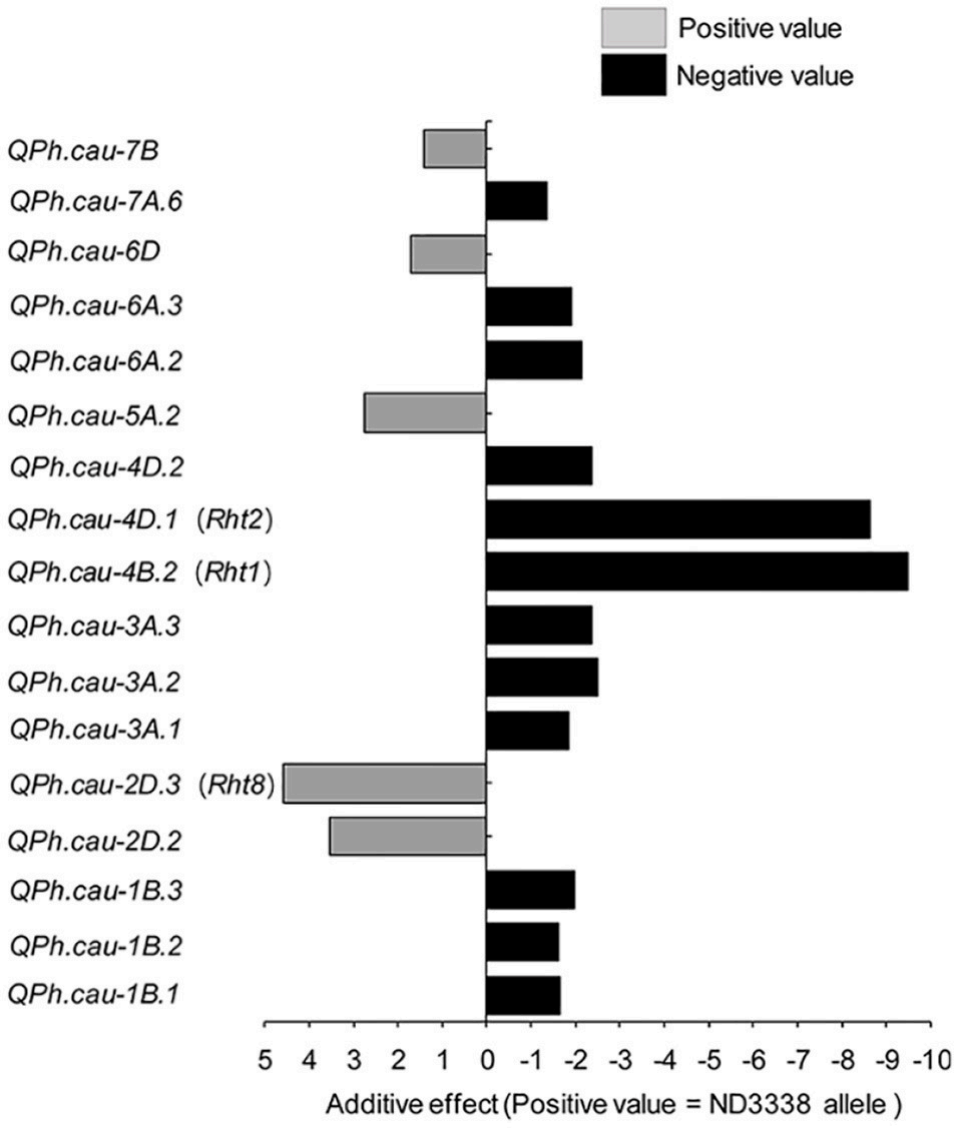
Stable QTL effects for plant height (PH) in the ND3338/JD6 DH population. Positive (+) and negative (−) values in the analysis of BLUP data are used to distinguish the additive effects of ND3338 and JD6 alleles in the DH population.

For PH, a total of 33 QTL were identified across eight shared environments with a LOD score range of 2.52–64.37, explaining 0.12–46.02% of the variance (Tables S6, S7). Of these QTL, 17 stable QTL were located on chromosomes 1B, 2D, 3A, 4B, 4D, 5A, 6A, 6D, 7A, and 7B (Figure 4, Table S6). *QPh.cau-1B.1, QPh.cau-2D.2, QPh.cau-2D.3, QPh.cau-3A.1, QPh.cau-3A.3, QPh.cau-4B.2, QPh.cau-4D.1*, and *QPh.cau-6A.2* were repeatedly identified in all eight individual environments and in the analysis of BLUP data, together explaining more than 95% of the observed variation of PH (Figure 4, Table S6). The positive effect alleles were contributed by both parents, ND3338 and JD6. Moreover, based on gene diagnostic markers, the three major QTL (*QPh.cau-4B.2, QPh.cau-4D.1*, and *QPh.cau-2D.3*) were coincident with previously identified reduced height (*Rht*) genes *Rht1, Rht2*, and *Rht8*, respectively (Figure 4). Using the gene specific markers for *Rht1, Rht2*, and Xgwm261 for *Rht8*, 188 DH lines formed eight haplotypes with different allele combinations (Table 6). Comparing to the Haplotype 1 without three dwarfing genes, Haplotypes 2, 3, and 4 with one dwarfing gene, Haplotypes 5, 6, and 7 with both dwarfing genes and Haplotype 8 with triple dwarfing genes reduced PH by 14.06, 17.27, 17.99, 23.11, 27.13, 35.91, and 46.74%, respectively (Table 6).

**Table 6:** Comparison of yield-related trait means (± standard deviation) among different haplotypes with *Rht-B1, Rht-D1*, and *Rht8* based on the adjusted mean (BLUP) values of eight shared environments and heat susceptibility index of thousand grain weight (HSITGW) in both E11 and E12 locations in the ND3338/JD6 doubled haploid (DH) population.

**Group^a^**	***Rht-B1***	***Rht-D1***	***Rht8***	**PH_BLUP^b^**	**SPP_BLUP^b^**	**GNS_BLUP^b^**	**TGW_BLUP^b^**	**GWS_BLUP^b^**	**HSITGW_E11^b^**	**HSITGW_E12^b^**
Hap-1 (24)	*Rht-B1a*	*Rht-D1a*	*Rht8b*	90.23 ± 10.07^a^	9.59 ± 0.71^cd^	41.20 ± 3.32^d^	50.83 ± 3.98^a^	2.18 ± 0.13^bc^	0.81 ± 0.30^c^	0.91 ± 0.15^b^
Hap-2 (16)	*Rht-B1a*	*Rht-D1a*	*Rht8c*	77.54 ± 12.32^b^	9.42 ± 0.95^d^	44.20 ± 5.58^abc^	50.38 ± 4.34^a^	2.32 ± 0.34^a^	1.06 ± 0.25^ab^	0.98 ± 0.20^ab^
Hap-3 (28)	*Rht-B1b*	*Rht-D1a*	*Rht8b*	74.65 ± 6.79^b^	9.67 ± 0.60^cd^	46.59 ± 3.72^ab^	47.32 ± 3.98^bc^	2.29 ± 0.15^ab^	1.21 ± 0.32^a^	1.06 ± 0.14^a^
Hap-4 (29)	*Rht-B1a*	*Rht-D1b*	*Rht8b*	74.00 ± 8.30^bc^	10.08 ± 0.91^bc^	44.08 ± 4.11^bc^	51.12 ± 2.96^a^	2.32 ± 0.18^a^	0.92 ± 0.35^bc^	0.96 ± 0.17^ab^
Hap-5 (36)	*Rht-B1a*	*Rht-D1b*	*Rht8c*	69.38 ± 7.76^cd^	9.95 ± 0.66^bc^	44.89 ± 4.88^abc^	49.51 ± 4.60^ab^	2.30 ± 0.22^ab^	0.96 ± 0.26^bc^	1.01 ± 0.14^ab^
Hap-6 (14)	*Rht-B1b*	*Rht-D1a*	*Rht8c*	65.75 ± 7.47^d^	9.63 ± 0.51^cd^	47.02 ± 4.58^a^	46.38 ± 3.38^c^	2.23 ± 0.16^abc^	1.05 ± 0.27^ab^	1.04 ± 0.14^a^
Hap-7 (18)	*Rht-B1b*	*Rht-D1b*	*Rht8b*	57.83 ± 8.18^e^	10.39 ± 0.74^ab^	46.91 ± 3.53^ab^	43.81 ± 3.55^d^	2.14 ± 0.14^c^	1.11 ± 0.29^ab^	1.01 ± 0.19^ab^
Hap-8 (23)	*Rht-B1b*	*Rht-D1b*	*Rht8c*	48.06 ± 4.75^f^	10.65 ± 0.84^a^	42.22 ± 4.05^cd^	43.30 ± 4.33^d^	1.90 ± 0.18^d^	1.00 ± 0.42^abc^	1.01 ± 0.14^ab^

a *The numerals within the brackets indicate the number of DH lines with different haplotypes in the ND3338/JD6 doubled haploid (DH) population*.

b *Trait abbreviations: plant height, (PH); spikes per plant, (SPP); grain number per spike, (GNS); thousand grain weight, (TGW) and grain weight per spike, (GWS). Different letters are used to indicate means that differ significantly (P < 0.05, LSD test)*.

### Heat susceptibility index of TGW (HSITGW)

The analysis for the HSI was conducted to detect QTL directly related to stress performance traits. Using composite interval mapping, we detected 3 and 6 QTL for the HSITGW in E11 and E12 locations, respectively (Table S8). Individual QTL explained from 4.84% to 14.29% of the phenotypic variance for the HSITGW, and both parents contributed favorable alleles. *QHsitgw.cau-4B.2* was repeatedly detected in both locations, which explained 9.23–9.92% of the total variation and co-localized with a minor QTL (*QTgw.cau-4B.7*) for TGW (Figure 2, Tables 7, 8, Tables S7, S8). The parent JD6 contributed the positive allele, enhancing heat tolerance. In addition, the localization of *QHsitgw.cau-2D* on chromosome 2DS coincided with *QTgw.cau-2D.2*, explaining the highest observed variation (14.29%) for HSITGW in the E12 location (Tables S7, S8).

**Table 7:** Stable QTL contents of the DH lines with top 10 high-TGW in the ND3338/JD6 population based on AMMI biplot analysis across 12 environments.

**QTL&Traits&PC^a^**	**Environment^b^**	**Additive alleles^c^**	**Nearest marker**	**Top 10 high-TGW genotypes^d^**									
				**DH198**	**DH154**	**DH144**	**DH143**	**DH185**	**DH203**	**DH135**	**DH138**	**DH119**	**DH134**
*QTgw.cau-2A.3*	C	J	*BS00065865_51*	B	B	B	A	B	A	B	B	B	B
*QTgw.cau-4A.3*	C	J	*Excalibur_c11968_204*	B	B	B	A	B	B	B	A	B	B
*QTgw.cau-4B.1*	C	J	*BS00011510_51*	B	A	B	B	B	B	A	B	B	A
*QTgw.cau-4B.2*	C	J	*BobWhite_c20051_53*	–	B	B	B	B	B	A	B	B	A
*QTgw.cau-4B.3*	C	J	*BS00084904_51*	B	B	B	B	A	B	B	B	B	B
*QTgw.cau-4B.4*	C	J	*Kukri_c61940_259*	B	B	B	B	B	B	B	B	B	B
*QTgw.cau-2D.1*	C	N	*GENE-0641_239*	B	A	A	A	A	A	B	B	A	B
*QTgw.cau-2D.2*	C	N	*Kukri_c19540_425*	A	A	A	A	A	A	B	A	B	B
*QTgw.cau-5A.2*	C	N	*BS00021708_51*	B	A	A	A	A	A	A	B	A	A
*QTgw.cau-6A.4*	C	N	*RFL_Contig6053_2072*	A	A	A	A	A	A	B	A	B	B
*QTgw.cau-7A.2*	C	N	*Excalibur_c49272_174*	A	A	A	A	A	A	A	B	B	A
*QTgw.cau-7A.3*	C	N	*BS00098482_51*	–	A	A	A	A	–	A	B	B	A
*QTgw.cau-7A.4*	C	N	*IAAV6957*	A	A	A	A	A	A	A	B	A	A
*QHsitgw.cau-4B.2*	E11	N	*WMC652*	B	B	B	A	B	B	B	B	B	B
*QHsitgw.cau-4B.2*	E12	N	*GPW7390*	B	B	B	A	B	B	B	B	B	B
TGW_BLUP				61.52	59.02	58.29	56.64	56.48	56.27	56.22	55.65	55.54	55.32
HSITGW_E11				0.90	0.49	0.83	0.39	1.45	0.45	0.53	0.66	0.84	0.58
HSITGW_E12				0.89	0.97	0.78	0.80	1.01	0.99	0.95	0.84	0.94	1.03
PC1				1.06	1.40	0.36	0.06	0.69	0.02	0.25	0.88	1.08	−0.19
PC2				−0.01	0.05	0.89	−0.31	−0.08	0.62	−0.28	0.06	0.74	−0.42

a The adjusted mean (BLUP) values of eight shared environments for thousand grain weight (TGW). Heat susceptibility index of thousand grain weight (HSITGW) in both E11 and E12 locations. PC, principal component based on AMMI biplot analysis across 12 environments(E1–E12).

b C indicates the combined QTL analysis based on BLUP values of eight shared environments.

c “N” and “J” represent the increasing alleles from ND3338 and JD6, respectively.

d *Different colors of “A” and “B” represent different genotypes from ND3338 and JD6, respectively*.

**Table 8:** Stable QTL contents of the DH lines with top 10 low-TGW in the ND3338/JD6 population based on AMMI biplot analysis across 12 environments.

**QTL&Traits&PC ^a^**	**Environment ^b^**	**Additive alleles ^c^**	**Nearest marker**	**Top 10 low-TGW genotypes^d^**									
				**DH93**	**DH39**	**DH79**	**DH201**	**DH1**	**DH101**	**DH65**	**DH68**	**DH94**	**DH62**
*QTgw.cau-2A.3*	C	J	*BS00065865_51*	A	B	B	A	A	B	B	B	A	B
*QTgw.cau-4A.3*	C	J	*Excalibur_c11968_204*	B	A	A	B	A	A	B	A	A	B
*QTgw.cau-4B.1*	C	J	*BS00011510_51*	A	A	A	A	A	A	A	A	A	A
*QTgw.cau-4B.2*	C	J	*BobWhite_c20051_53*	A	B	A	A	A	A	A	A	A	A
*QTgw.cau-4B.3*	C	J	*BS00084904_51*	A	A	A	A	A	A	A	A	A	A
*QTgw.cau-4B.4*	C	J	*Kukri_c61940_259*	A	B	A	A	A	A	A	A	A	A
*QTgw.cau-2D.1*	C	N	*GENE-0641_239*	B	A	B	B	B	B	B	B	B	B
*QTgw.cau-2D.2*	C	N	*Kukri_c19540_425*	A	B	B	B	B	B	B	B	A	B
*QTgw.cau-5A.2*	C	N	*BS00021708_51*	A	B	B	B	B	A	B	A	A	B
*QTgw.cau-6A.4*	C	N	*RFL_Contig6053_2072*	B	B	A	A	A	B	B	B	B	B
*QTgw.cau-7A.2*	C	N	*Excalibur_c49272_174*	B	B	B	A	A	B	B	B	–	B
*QTgw.cau-7A.3*	C	N	*BS00098482_51*	B	B	B	A	A	B	B	B	B	B
*QTgw.cau-7A.4*	C	N	*IAAV6957*	A	B	A	A	A	B	A	B	A	A
*QHsitgw.cau-4B.2*	E11	N	*WMC652*	A	B	B	A	B	A	A	A	A	A
*QHsitgw.cau-4B.2*	E12	N	*GPW7390*	A	B	B	A	B	A	A	A	A	A
TGW_BLUP				40.26	39.58	39.14	38.99	39.20	38.14	37.62	37.31	36.57	36.72
HSITGW_E11				1.10	0.96	0.43	0.51	0.70	1.51	1.12	1.31	1.25	0.70
HSITGW_E12				1.01	1.00	1.23	1.03	0.97	1.08	1.13	1.05	0.96	1.12
PC1				−0.76	−0.86	−0.79	−1.06	−0.77	−0.94	−1.06	−0.90	−0.66	−0.73
PC2				0.44	0.48	−0.02	−0.34	0.49	0.26	−0.04	0.41	1.09	−0.01

a The adjusted mean (BLUP) values of eight shared environments for thousand grain weight (TGW). Heat susceptibility index of thousand grain weight (HSITGW) in both E11 and E12 locations. PC: principal component based on AMMI biplot analysis across 12 environments (E1–E12).

b C indicates the combined QTL analysis based on BLUP values of eight shared environments.

c “N”and “J” represent the increasing alleles from ND3338 and JD6, respectively.

d *Different colors of “A” and “B” represent different genotypes from ND3338 and JD6, respectively*.

## Discussion

### Phenotypic variations in response to environments

**Figure 5:**
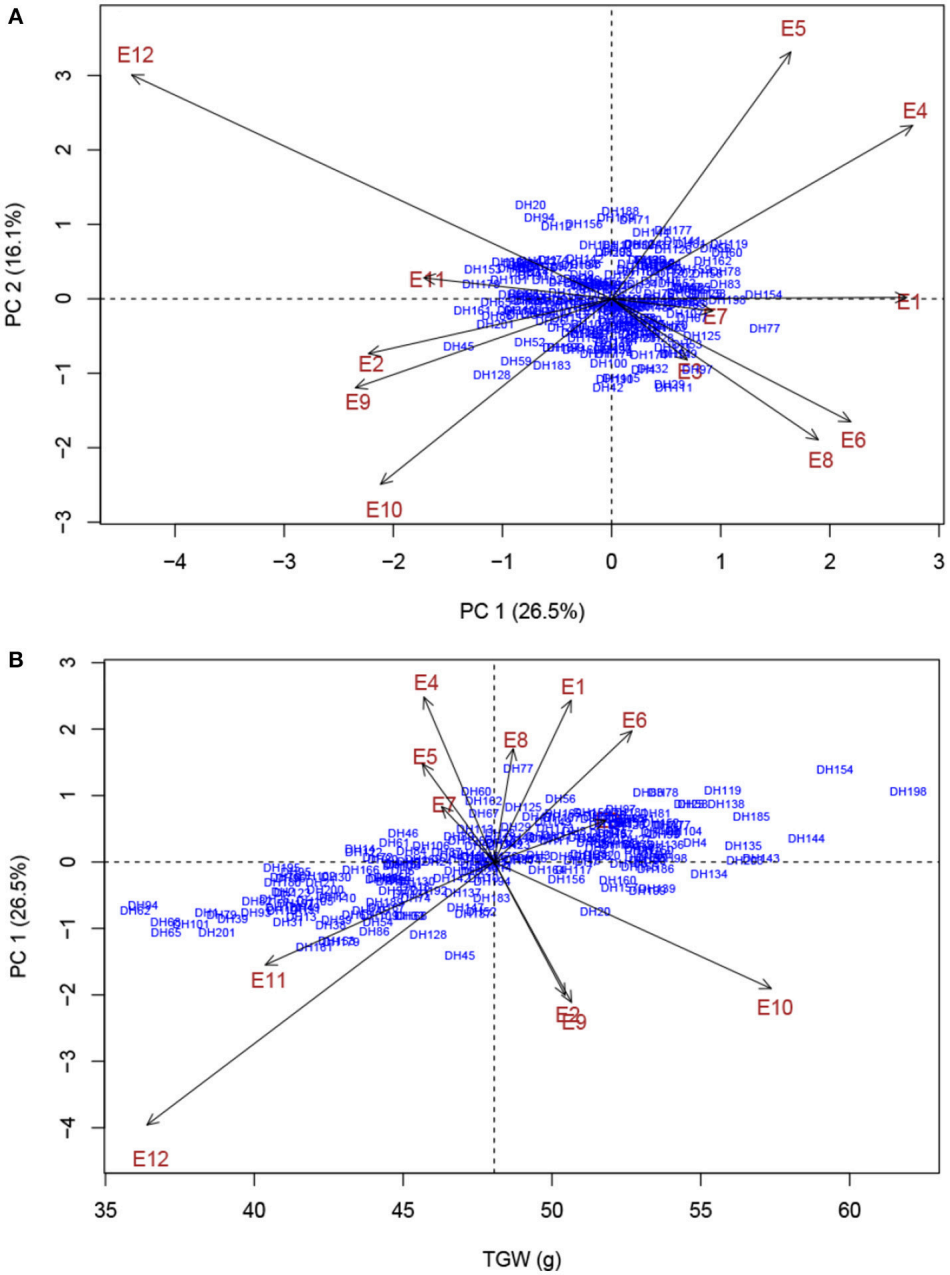
AMMI biplot analysis of thousand grain weight (TGW) across 12 experimental trails for environmental variability and genotypic stability. **(A)** AMMI biplot for the first principal component of the interaction (PC 1) × second principal component of the interaction (PC 2) for TGW of 203 DH genotypes evaluated in 12 environments. **(B)** AMMI biplot for main effects and genotype by environment interaction for TGW of 203 DH genotypes evaluated in 12 environments. Codes for the environments are explained in Table 1.

Phenotypic characterization of yield-related traits over multi-environment field trials is essential for assessing trait stability across environments and contributes to accurate identification of stable genomic regions (Ogbonnaya et al., 2017). With rising global temperature and climate change, heat stress is becoming an increasingly severe constraint on wheat production in many parts of the world (Paliwal et al., 2012; Ni et al., 2017). In the present study, the two parents, ND3338 and JD6, are elite winter wheat varieties from the Northern Winter Wheat Zone in China, and Beijing and Shijiazhuang sites are optimum growing zones. The Sanyuan site is located in the Yellow and Huai River Valleys Facultative Wheat Zone. The Linfen and Urumqi sites were hotspot regions of hot and dry winds during the grain filling period (Liu B. et al., 2014; Yang et al., 2017). Moreover, AMMI biplot based on the population means of five yield-related traits across eight shared sites depicted the environmental difference and genotype by environment interaction (Figure 1), indicating that the identified QTL for yield-related traits showed stability in a wide range of environments. Two additional experiments under high environmental temperatures due to a delayed planting date were conducted to further evaluate the performance of the parents and the DH population under heat stress. Consequently, late-sown environments in Linfen and Sanyuan with a maximum temperature >35°C during the grain filling stage resulted in 20.09–36.36% reduction of grain weight compared to the control, which is consistent with the results of AMMI analysis (Figure 5).

### Consensus and novel QTL compared with previous research

Grain yield and yield-related traits are complex quantitative traits with polygenic inheritance and are highly affected by environments. The identification of QTL associated with yield-related traits across diverse environments on different wheat chromosomes has previously been reported (Quarrie et al., 2005; Zhang et al., 2010; Liu G. et al., 2014; Gao et al., 2015; Wu et al., 2015; Würschum et al., 2015; Nadolska-Orczyk et al., 2017; Shi et al., 2017). The 50 stable QTL governing yield-related traits in the present work were mainly distributed on chromosomes 1B, 2A, 2B, 2D, 3A, 4A, 4B, 4D, 5A, 6A, 6B, 6D, 7A, and 7B in a non-random manner (Table 5, Table S6).

In this study, three major stable QTL (*QPh.cau-4B.2, QPh.cau-4D.1*, and *QPh.cau-2D.3*) for PH were detected on chromosomes 4BS, 4DS, and 2DS at positions within the three dwarfing genes *Rht-B1, Rht-D1*, and *Rht8*, respectively, which agrees with results from previous studies (Peng et al., 1999; Zhang et al., 2006; Gao et al., 2015). A stable locus (*QPh.cau-5A.2*) that had an effect on PH was found on chromosome 5A at 86.70–95.00 cM; similarly, the dwarfing gene *Rht9* was previously reported to be located on chromosome 5AL (Wu et al., 2010). Furthermore, a minor but stable PH QTL, *QPh.cau-6A.2*, was detected across all environments in this study. In parallel, Tian et al. (2017) also found a major quantitative trait locus (*QPH.caas-6A*) in a similar region on chromosome 6A, which was designated *Rht24*. After that, Würschum et al. (2017) assessed the relevance of *Rht24* using an association mapping approach based on a large panel of 1,110 winter wheat cultivars, suggesting that *Rht24* was an important *Rht* gene of commercial relevance in worldwide wheat breeding. To the best of our knowledge, the stable QTL *QPh.cau-1B.2, QPh.cau-2D.2, QPh.cau-3A.2, QPh.cau-3A.3, QPh.cau-4D.2, QPh.cau-6A.3*, and *QPh.cau-7B* were likely novel QTL for PH owing to the high-density integrated genetic linkage map and special performance of the genetic background between ND3338 and JD6. These novel loci displayed relatively smaller additional effects compared with *Rht1, Rht2*, and *Rht8*; hence, near isogenic lines are ideally required in future work to determine their effects on PH and other agronomic traits dependent on the environment.

For GWS and yield components, we detected a QTL cluster for TGW and GWS located in the interval 112.90–133.80 cM on chromosome 2AL and a QTL cluster for GNS and GWS located in the interval 111.60–131.90 cM on chromosome 2B; similar genomic regions were previously reported to harbor QTL for GNS, TGW, and GWS using the different recombinant inbred line (RIL) populations (Cui et al., 2014; Liu G. et al., 2014; Gao et al., 2015.) The genomic regions with the highest numbers of stable QTL for yield components were on group-4 chromosomes near the Green Revolution genes *Rht1* and *Rht2*, which agrees with results from earlier reports (Zhang et al., 2013; Liu G. et al., 2014). Furthermore, the stable QTL for GNS and TGW were found on chromosome 5A; and previous studies also identified stable loci for GNS and TGW at the similar interval based on linkage analysis and GWAS approach (Cui et al., 2014; Gao et al., 2015; Wu et al., 2015; Ogbonnaya et al., 2017). Likewise, another QTL-rich region associated with GNS, GWS, and TGW was identified on chromosome 7A in this study; consistently, Quarrie et al. (2006) reported the co-localized QTL cluster for yield and yield-related traits on chromosomes 7A in the Chinese Spring/SQ1 DH population under stressed and non-stressed conditions, some of which were also validated using near isogenic lines.

In this study, we identified 8 QTL for the HSI of TGW. Among these QTL, *QHsitgw.cau-6D* was identified on chromosome 6DL in the Sanyuan location, which is consistent with the study of Mason et al. (2010), which identified QTL for the HSI of kernel weight in a similar position using a RIL population derived from a cross between the heat-tolerant cultivar “Halberd” and heat-sensitive cultivar “Cutter.” Notably, the locus *QHsitgw.cau-4B.2* was detected in the two heat-stressed trials and was located on chromosome 4BL in the interval 115.80–123.10 cM, spanning 14.28 Mb (4B: 620058668–4B: 634340181) in physical position (Tables S4, S8). Moreover, IAAV5323 is a SNP in the confidence interval within the protein coding region of the annotated gene *TraesCS4B01G340600* encoding Zinc finger protein CONSTANS (Table S10), which is known to be involved in the regulation of multiple processes, including flowering time, hormone metabolism, and biotic and abiotic stresses (Griffiths et al., 2003; Weng et al., 2014; Kiss et al., 2017). Thus, we postulate that *TraesCS4B01G340600* is a possible candidate gene for the *QHsitgw.cau-4B.2* QTL. Moreover, to the best of our knowledge, *QHsitgw.cau-4B.2* is a novel stable QTL for the HSI of TGW, and diagnostic molecular markers can be developed and deployed within breeding programs. Additionally, the SNP maker IACX4386 for *QHsitgw.cau-1A* corresponded to the gene *TraesCS1A01G285000* encoding a 70-kDa heat shock protein; this result merits further investigation (Table S10).

### QTL/genes controlling PH showed pleiotropic effects on yield components

PH is an important agronomic trait in wheat, and wheat yield increases during the Green Revolution were achieved through the introduction of reduced height (*Rht*) dwarfing genes (Hedden, 2003; Zhang et al., 2006). QTL mapping in previous studies confirmed that PH is a complex trait controlled by the few major *Rht* loci and by minor QTL (Wu et al., 2010; Griffiths et al., 2012; Würschum et al., 2015, 2017; Tian et al., 2017). In this study, three major stable QTL (*QPh.cau-4B.2, QPh.cau-4D.1*, and *QPh.cau-2D.3*) for PH corresponded to *Rht1, Rht2*, and *Rht8*. Haplotype analysis revealed that *Rht* loci exhibited pleiotropic architecture, which affects not only PH but also yield component traits (Table 6). Significant differences were detected between Haplotype 1 and Haplotype 3 for PH, GNS, and TGW with the BLUP values. The dwarf gene Haplotype 2 and Haplotype 3 showed pleiotropic effects on PH, GNS, and GWS compared with Haplotype 1 (Table 6). Furthermore, Haplotype 8 exhibited the lowest PH, TGW, and GWS but the most SPP, indicating that shorter plants lead to the reduction of total biomass yield. Notably, for GNS, Haplotype 8 displayed no significant difference from Haplotypes 1, 2, 4, and 5 (Table 6). Additionally, previous research found that the two *Rht-1* semi-dwarfing genes only improved yields under optimal conditions, whereas tall isogenic lines without *Rht-B1b* or *Rht-D1b* yielded more than their *Rht-1b* carrying counterparts in biotic and abiotic stresses environments (Zhang et al., 2013; Würschum et al., 2017). Similarly, there is a significant difference in thermotolerance between Haplotype 1 and Haplotype 3 in our study (Table 6). Therefore, the choice of the *Rht* loci best suited for achieving the desired plant stature must account for pleiotropic effects on yield components in different geographic and climatic regions.

### Co-location of yield components revealed significant tradeoffs between TGW and GNS on chromosome 4A

TGW and GNS are two crucial but counteracting determinants of wheat grain yield (Quarrie et al., 2006; Schulthess et al., 2017; Zhai et al., 2017). In the present study, we identified two stable QTL on chromosome 4A controlling TGW (*QTgw.cau-4A.3*) and GNS (*QGns.cau-4A.4*) with superior alleles coming from the opposite parent (Table 5), which exhibited a strong pleiotropic trade-off between TGW and GNS, consistent with phenotypic correlation analysis (Table 4). For TGW, QTL in the same region of chromosome 4AL were reported by Cui et al. (2016) and Gao et al. (2015); however, to the best of our knowledge, this is the first report about stable locus *QTgw.cau-4A.3* for TGW under heat-stressed conditions. For GNS, interestingly, Cui et al. (2017) also identified a major stable QTL for kernel number per spike (KNPS) in 10 environments using a Wheat660K SNP array-derived high-density genetic map, and the overlapping confidence intervals spanned 3.23 Mb (4A: 680398739–4A: 683638403) in physical position, which is consistent with our results (4A: 632020468–4A: 684998591). Moreover, a QTL cluster for TKW and KNPS positioned in the interval 136.80–157.30 cM on chromosome 4AL corresponded to a similar physical interval (4A: 632864778–4A: 688093018) detected by Gao et al. (2015) using the Zhou 8425B/Chinese Spring RIL population, and the positive alleles increasing TGW and GNS were all contributed by Zhou 8425B, showing no negative pleiotropic trade-off. Hence, these coincidences confirmed the authenticity of this locus, which should be subjected to fine mapping and map-based cloning in the future. Only these techniques can distinguish between whether these loci have a pleiotropic effect or are closely linked. Additionally, we performed corresponding gene annotations and synteny analyses with rice genomes with the Chinese Spring IWGSC RefSeq v1.0 based on the SNP marker flanking sequences in the confidence interval (Table S9).

### Dissection of a “QTL-hotspot” region on chromosome 4B was useful in MAS for grain weight

Generally, at least three QTL for yield-related traits were identified on every chromosome in our study with chromosome 4B having the most QTL (Tables S6, S7). Thus, the shared genomic region with a pleiotropic effect or tightly linked loci affecting two or more traits on chromosome 4B is referred to as a “QTL-hotspot” region. Based on the positions of stable QTL for TGW, the “QTL-hotspot” region on chromosome 4B was artificially divided into three parts: region 4B.1, region 4B.2, and region 4B.3 (Figure 2). For region 4B.1, the stable QTL controlling TGW (*QTgw.cau-4B.1* and *QTgw.cau-4B.2*) and SPP (*QSpp.cau-4B.3* and *QSpp.cau-4B.4*) with favored alleles coming from the opposite parent were detected, exhibiting strong TGW-SPP tradeoffs, and this result was consistent with the phenotypic negative correlation. Similarly, Chen et al. (2014) identified a stable QTL for TGW on chromosome 4BS with the same SNP makers using the Shannong 01-35/gaocheng9411 RIL population, and it was considered to be a novel QTL for TGW in their results. For region 4B.2, the stable QTL (*QTgw.cau-4B.3*) was co-localized with one QTL for PH (*QPh.cau-4B.2*), and increasing alleles all came from JD6. Meanwhile, it was corroborated as a pleiotropic consequence of the dwarfing gene *Rht1* in the present study, consistent with the results previously published in wheat (Liu G. et al., 2014; Schulthess et al., 2017). For region 4B.3, we found that the stable QTL (*QTgw.cau-4B.4*) was also constitutively expressed in two heat-stressed environments, and more importantly, it displayed no negative pleiotropic association with the other two yield component traits (GNS and SPP). Although previous research also reported the detection of QTL for grain weight or grain dimensions on chromosome 4B through linkage analysis and genome-wide association analysis (Quarrie et al., 2005; Liu G. et al., 2014; Huang et al., 2015; Wu et al., 2015; Zanke et al., 2015; Cui et al., 2016; Kumar et al., 2016), to the best of our knowledge, this is the first report on the dissection of the QTL cluster on chromosome 4B for TGW. We speculate that the “QTL-hotspot” region on chromosome 4B may have experienced artificial selection during past breeding practice and played an important role in grain yield determination and improvement, especially on grain weight. Thus, dissection of a “QTL-hotspot” region on chromosome 4B was useful for future map-based cloning and MAS-based QTL pyramiding for grain weight.

### QTL combinations for TGW and HSITGW explored the value of breeding

To explore the effects of different QTL combinations on grain weight, the favored or unfavored allele effects from ND3338 and JD6 were simulated. The patterns of the relationships were similar, where TGW increased gradually along with increases in favored alleles from ND3338 or JD6 (Figure 3). Outstandingly, favored alleles from JD6 showed higher R-squared values in linear regression analysis (*R*^2^ = 0.53) than the values for favored alleles from ND3338 (*R*^2^ = 0.12), consistent with the total phenotypic variation explained by QTL analysis (Tables S6, S7). Moreover, AMMI analysis was conducted to rank genotypes based on TGW across 12 environments, and the top 10 high-TGW genotypes and top 10 low-TGW genotypes were identified to determine the genetic composition of stable QTL for TGW. Among the 13 stable QTL for TGW, top 10 high-TGW genotypes possess at least 7 favorable alleles from ND3338 or JD6, whereas top 10 low-TGW genotypes have no more than 5 favorable alleles (Table 7). Moreover, top 10 high-TGW genotypes all have the major robust QTL (*QTgw.cau-4B.4*) that harbors favored alleles from JD6 (Table 7), whereas the stable QTL (*QTgw.cau-4B.1* and *QTgw.cau-4B.3*) that harbor unfavorable alleles from ND3338 were all observed in top 10 low-TGW genotypes (Table 8). Additionally, 9 out of the top 10 high-TGW genotypes for *QHsitgw.cau-4B.2* carried the favored alleles that increase thermotolerance, and only three out of the top 10 low-TGW genotypes carried the favored alleles (Tables 7, 8). Furthermore, the top 10 high-TGW genotypes exhibited relatively more stable performance across all seasons compared to the top low-TGW genotypes based on the principal component values (PC1 and PC2). Taken together, our results provide a basis for developing new heat-tolerant wheat varieties with high yield stability via a molecular design breeding strategy to fix good additive alleles.

## Conclusion

Overall, this study identified a total of 226 QTL controlling five yield-related traits (i.e., PH, SPP, GNS, TGW, and GWS) and the heat susceptibility index (HSI) in Nongda3338/Jingdong6 DH population across 12 different field trials with normal and late sowing heat stress conditions. Of these, 39 stable QTL for PH, SPP, GNS, TGW, and GWS were mapped within 12 genomic regions with corresponding physical intervals of Chinese Spring RefSeq v1.0 sequence on chromosomes 1B, 2A, 2B, 2D, 3A, 4A, 4B, 4D, 5A, 6A, and 7A. Three QTL *QPh.cau-4B.2, QPh.cau-4D.1* and *QPh.cau-2D.3* corresponded to dwarfing genes *Rht1, Rht2*, and *Rht8*, which had the pleiotropic effect on yield component traits. A QTL-hotspot region on chromosome 4B for grain weight and a novel QTL for HSITGW on chromosome 4BL were detected. These results will contribute to our understanding of the genetic basis of yield-related traits, and could be used to improve grain yield and in wheat through MAS breeding after validation.

## Author contributions

HP: conceived the project; PG, LL, and LJ: carried out experiments; MK, JZ, TL, and YZ: participated in field trials; PG: analyzed experimental results; PG and HP: wrote the manuscript; MX, ZH, YY, ZN, and QS: helped to revise the manuscript. All authors have read and approved the final manuscript.

### Conflict of interest statement

The authors declare that the research was conducted in the absence of any commercial or financial relationships that could be construed as a potential conflict of interest.

## Abbreviations

AMMI: additive main effects and multiplicative interactions
BLUP: best linear unbiased prediction
DH: doubled haploid
GNS: grain number per spike
GWS: grain weight per spike
HSITGW: heat susceptibility index of thousand grain weight
MAS: marker-assisted selection
PH: plant height
QTL: quantitative trait locus
Rht: reduced height
RIL: recombinant inbred line
SNP: single-nucleotide polymorphism
SPP: spikes per plant
SSR: single sequence repeats
STS: sequence-tagged sites
TGW: thousand grain weight.
