# Supplementary figures and images for: Global QTL Analysis Identifies Genomic Regions on Chromosomes 4A and 4B Harboring Stable Loci for Yield-Related Traits Across Different Environments in Wheat (Triticum aestivum L.)
Source: Front Plant Sci. 2018 Apr 25;9:529. doi: 10.3389/fpls.2018.00529 (PMC5996883; doi:10.3389/fpls.2018.00529)

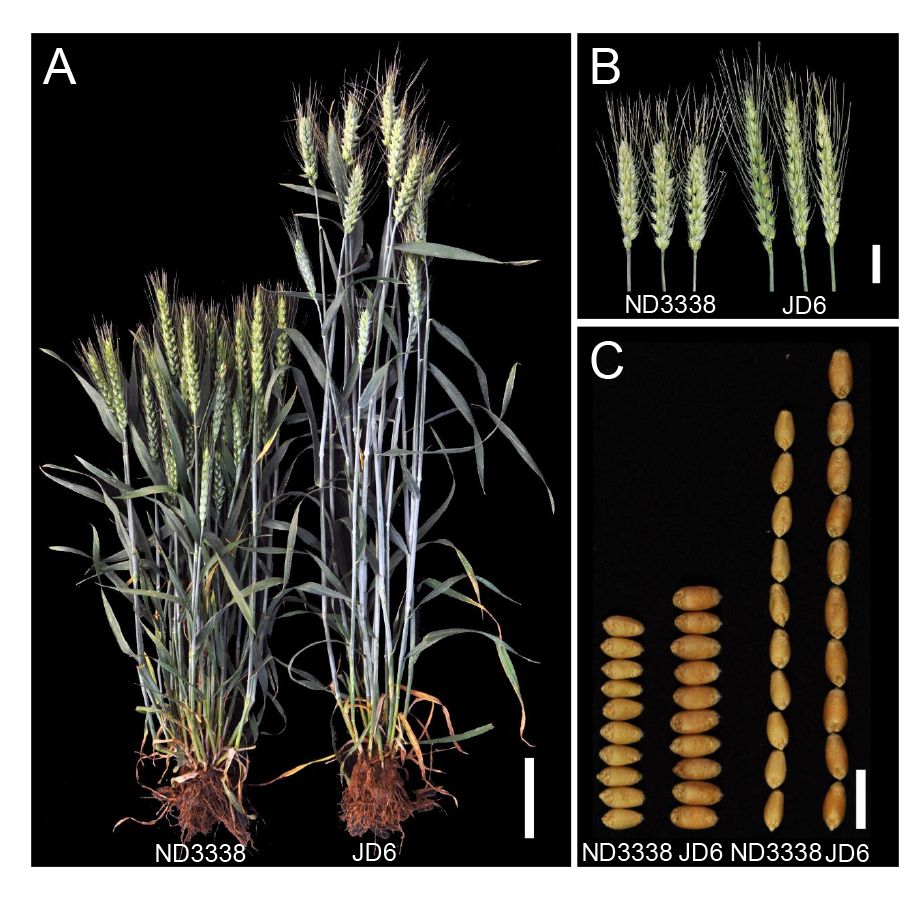

Supplement: Supplementary file 2 [file Image_1.tif]

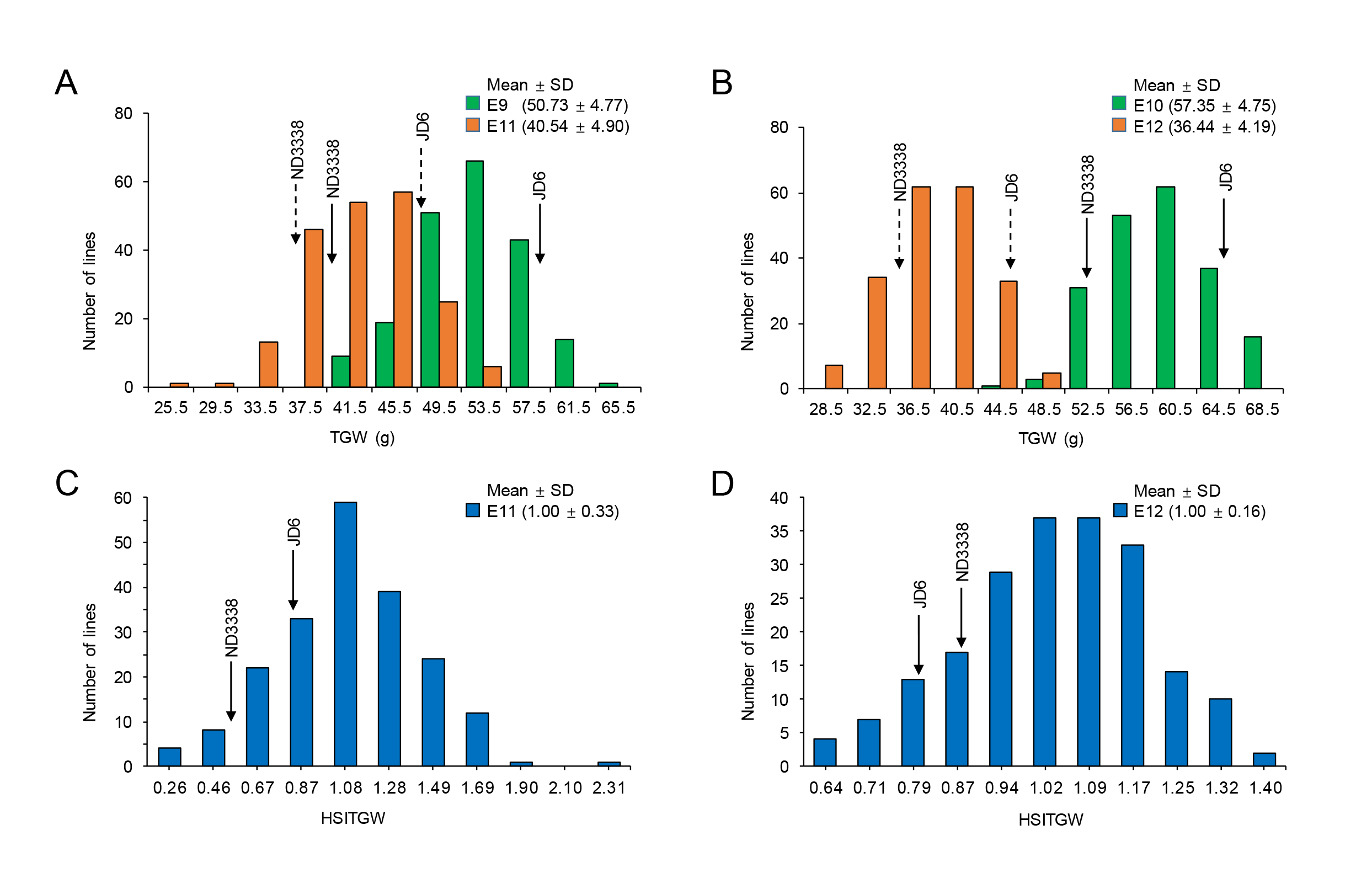

Supplement: Supplementary file 3 [file Image_2.tif]
